# Aberrant expression of NEDD4L disrupts mitochondrial homeostasis by downregulating CaMKKβ in diabetic kidney disease

**DOI:** 10.1186/s12967-024-05207-6

**Published:** 2024-05-16

**Authors:** Fei Han, Shi Wu, Ya Dong, Yanjie Liu, Bei Sun, Liming Chen

**Affiliations:** NHC Key Laboratory of Hormones and Development, Tianjin Key Laboratory of Metabolic Diseases, Chu Hsien-I Memorial Hospital Tianjin and Institute of Endocrinology, Tianjin Medical University, Tianjin, 300134 China

**Keywords:** Diabetic kidney disease, Mitochondrial homeostasis, NEDD4L, CaMKKβ

## Abstract

Disturbance in mitochondrial homeostasis within proximal tubules is a critical characteristic associated with diabetic kidney disease (DKD). CaMKKβ/AMPK signaling plays an important role in regulating mitochondrial homeostasis. Despite the downregulation of CaMKKβ in DKD pathology, the underlying mechanism remains elusive. The expression of NEDD4L, which is primarily localized to renal proximal tubules, is significantly upregulated in the renal tubules of mice with DKD. Coimmunoprecipitation (Co-IP) assays revealed a physical interaction between NEDD4L and CaMKKβ. Moreover, deletion of NEDD4L under high glucose conditions prevented rapid CaMKKβ protein degradation. In vitro studies revealed that the aberrant expression of NEDD4L negatively influences the protein stability of CaMKKβ. This study also explored the role of NEDD4L in DKD by using AAV-shNedd4L in db/db mice. These findings confirmed that NEDD4L inhibition leads to a decrease in urine protein excretion, tubulointerstitial fibrosis, and oxidative stress, and mitochondrial dysfunction. Further in vitro studies demonstrated that si-Nedd4L suppressed mitochondrial fission and reactive oxygen species (ROS) production, effects antagonized by si-CaMKKβ. In summary, the findings provided herein provide strong evidence that dysregulated NEDD4L disturbs mitochondrial homeostasis by negatively modulating CaMKKβ in the context of DKD. This evidence underscores the potential of therapeutic interventions targeting NEDD4L and CaMKKβ to safeguard renal tubular function in the management of DKD.

**Supplementary Information:**

The online version contains supplementary material available at 10.1186/s12967-024-05207-6.

## Introduction

Diabetic kidney disease (DKD) is deemed to be the most prevalent and serious long-term complication of diabetes [1]. Its clinical symptoms include the onset of proteinuria, and a subsequent decrease in glomerular filtration that correlates with glomerulosclerosis [2]. The theory regarding the tubular aspect of DKD has gained prominence in the past decade, underscoring the critical role of proximal tubule dysfunction in the manifestation of albuminuria related to diabetic tubulopathy [3]. Proximal tubular cells are rich in mitochondria, which provide the energy required for tubular secretion and reabsorption [4, 5]. The wellness of mitochondria is vital for facilitating appropriate oxidative phosphorylation and ATP production. Notably, in DKD patients and corresponding mouse models, specific disruptions in mitochondrial homeostasis within proximal tubules have been identified [6, 7]. Hence, enhancing mitochondrial homeostasis in renal proximal tubular cells is likely a promising therapeutic and preventive measure for DKD.

Mitochondria are exceedingly dynamic organelles that constantly undergo fusion/fission and turnover through mitochondrial biogenesis and mitophagy [8]. Mitochondrial fusion reduces stress by joining partially damaged mitochondrial contents as a form of complementation, whereas fission is necessary to clear damaged mitochondria via the segregation of dysfunctional mitochondria [9]. However, excessive mitochondrial fission can lead to mitochondrial fragmentation, impair the electron transport chain and cause abnormal accumulation of mitochondrial ROS (mito-ROS). This accumulation of mito-ROS can contribute to mitochondrial dysfunction in DKD [10, 11].

Calcium ions (Ca^2+^), which serve as essential and consistent second messengers, also support mitochondrial function [12]. It directly affects CaMKKβ (calmodulin-dependent protein kinase β), a standard downstream molecule in Ca^2+^ signaling, with its activity dependent on Ca^2+^ concentration [13]. Furthermore, CaMKKβ has the capacity to phosphorylate and stimulate 5’ AMP-activated kinase (AMPK) [14], recognized for its vital role in regulating cell energy metabolism, responding to oxidative stress, and monitoring mitochondrial operations [15]. The CaMKKβ/AMPK signaling pathway has proven to significantly regulate the mitochondrial stability of tubular epithelial cells [16]. Its activation counters DKD by modulating apoptosis and autophagy in endothelial cells and podocytes [17, 18]. Nevertheless, the complete impact of the CaMKKβ/AMPK pathway on the regulation of diabetic tubular epithelial cells remains to be fully elucidated.

Previous studies have shown that the attenuation of CaMKKβ could contribute to the pathogenesis of DKD [17, 18]. However, the precise underlying mechanism remains unclear. NEDD4L, a ligase enriched in the kidney, pancreas, and prostate, is primarily expressed in renal tubular epithelial cells. Existing data indicate that NEDD4L is deeply involved in multiple calcium-related pathways [19, 20]. Furthermore, NEDD4L-depleted cells displayed a heightened mitochondrial membrane potential and maintained a mitochondrial fusion status in response to metabolic stress [21]. In light of these findings, we postulate that NEDD4L potentially modulates mitochondrial homeostasis by influencing CaMKKβ activity in diabetic renal tubular epithelial cells.

In our recent study, we illustrated the potential role of NEDD4L in inducing mitochondrial dysfunction in diabetic proximal tubular epithelial cells. It was also established that the disruption of mitochondrial homeostasis in DKD could be attributed to the inhibition of CaMKKβ/AMPK signaling. Interestingly, our results suggest that NEDD4L targets the degradation of CaMKKβ, leading to the suppression of the CaMKKβ/AMPK signaling pathway and subsequent mitochondrial impairment in proximal tubular epithelial cells.

## Materials and methods

### Animal models

Eight-week-old male db/db mice and their non-diabetic db/m littermates were obtained from Jicui Bioscience Co. Ltd. (Jiangsu, China), and were used as the type 2 diabetes models. All mice were housed individually, subjected to a 12-hour light/dark cycle, given free access to water, and were nourished with a standard laboratory diet. Twenty of the db/db mice were given injections of either adeno-associated virus (AAV)-shNedd4l-EGFP or the control virus (Yuanjing Biosciences, Guangdong, China). The Animal Care Committee of Tianjin Medical University strictly regulated all animal experiments according to its established guidelines and regulations.

### Cell culture

Proximal tubular epithelial cells of human origin, HK-2, were procured from the Cell Bank of the Chinese Academy of Sciences, based in Shanghai, China, and subsequently cultured in DMEM medium, which was fortified with a 10% concentration of fetal bovine serum. The HK-2 cells were subjected to culture conditions that consisted of either a normal glucose concentration of 5.5 mM or a high glucose (HG) concentration of 25.5 mM over a period of 48 h. Additionally, 20.0 mM mannitol was used as an osmolality control. The cell transfection process was executed using the plasmids pcDNA3.1-CaMKKβ (FulenGen, Guangdong, China), Nedd4L-siRNA (Ribobio, Guangdong, China) and CaMKKβ-siRNA (Ribobio, Guangdong, China), along with the Lipofectamine 2000 reagent (Invitrogen, California, USA) in compliance with the guidelines provided by the manufacturer.

### Renal histological analysis

As previously described [22], renal histological examination was conducted. Briefly, protocols provided by the manufacturer were followed to stain kidney sections with Hematoxylin and Eosin (HE), Masson’s trichrome, and Periodic acid-Schiff (PAS). For immunohistochemical (IHC) analysis, kidney sections were blocked and incubated with primary antibodies. Subsequent incubation with secondary antibodies was performed before visualization using diaminobenzidine. The final step of counterstaining was performed with hematoxylin.

### Western blotting analysis

Total proteins or mitochondrial proteins were isolated, separated and transferred onto polyvinylidene fluoride membranes (Millipore, Boston, USA). The membranes were incubated overnight with primary antibodies. The primary antibodies and their respective dilutions were NEDD4L, p-AMPK, AMPK, DRP-1, and HSP60 (all with a dilution ratio of 1:1000, Cell Signaling Technology, Danvers, USA), CaMKKβ and MFN-2 (1:1000 dilution, Abcam, Cambridge, UK), and phospho-DRP1 (Ser616) (1:1000 dilution, Biorbyt, Cambridge, UK). Following this procedure, the sections were incubated with secondary anti-mouse/rabbit antibodies (Sungene Biotech, Tianjin, China). The protein bands were subsequently visualized using ECL Blotting Detection Reagents (Invigentech, California, USA). The final quantification of these blots was achieved using densitometry via the application of ImageJ software.

### Isolation of mitochondrial protein

The mitochondrial fractions were extracted with a Mitochondria/Cytosol Fractionation Kit (KeyGEN BioTECH, Jiangsu, China) according to the manufacturer’s instructions.

### Immunofluorescence (IF) staining

For IF staining of cells and tissues, the protocols used were identical to those used for IHC analysis. Following incubation with the primary antibody, the slides were subjected to serial incubation with goat anti-rabbit IgG/FITC at a concentration of 1:100 (Proteintech, Chicago, USA). Subsequently, the slides were imaged using an automated Leica DMI 4000 B inverted microscope equipped with a Leica DFC300 FX camera.

### Assessment of mitochondrial morphology

After several treatments, the viable cells were incubated with MitoTracker (Cell Signaling Technology, Danvers, USA) following the manufacturer’s guidelines. Subsequently, these cells were inspected via confocal microscopy.

### Albumin uptake analyses

The endocytosis assay was conducted according to a previously established procedure. In brief, HK2 cells were washed with warm Hanks’ balanced salt solution (without phenol red) and subsequently incubated with 0.5 mg/ml TRITC-bovine serum albumin (BSA) (Sigma-Aldrich, USA) for one-quarter of an hour at 37 °C. After incubation, six washes were performed on the cells using cold Hanks’ balanced salt solution (at 4 ℃), followed by lysis in PBS buffer. A confocal fluorescence microscope (Leica Microsystems, Germany) was used to detect the fluorescence within the lysate.

### Detection of ROS

To assess intracellular and mitochondrial ROS production, cells were incubated with H2DCFDA (Keygenbio, China) or Mitosox (Keygenbio, China), respectively, and examined by microscopy. Intracellular ROS production in kidney tissues was evaluated utilizing 4-mm-thick cryostat sections, which were then stained with dihydroethidium (DHE) acquired from EVERBRIGHT, USA.

### Co-immunoprecipitation (Co-IP)

The immunoprecipitation (IP) experiments were conducted in accordance with the methods previously reported [23]. Briefly, cells were harvested and lysed in IP lysis buffer, which consisted of 50 mM Tris (pH 7.4), 1 mM EDTA, 150 mM NaCl, 0.5% NP-40, and 1× protease inhibitor cocktail. Following an incubation period of one hour at 4 °C, the cell lysates were centrifuged at 14,000 × g for 10 min at 4 °C. Both NEDD4L and CaMKKβ were immunoprecipitated from 500 mg of the digitonin-solubilized proteins using Protein A/G PLUS-Agarose (Santa Cruz Biotechnology, CA, USA) according to the manufacturer’s guidelines. The immunocaptured proteins were then subjected to SDS‒PAGE/immunoblotting to analyze protein‒protein interactions.

### Statistical analyses

SPSS 19.0 software was used for statistical analysis. All the values are expressed as the mean ± SEMs. The data were analyzed using one-way analysis of variance (ANOVA) or the least significant difference (LSD) test. A value of *P* < 0.05 was considered to indicate statistical significance.

## Results

### Alterations in mitochondrial dynamics in renal proximal tubular cells of diabetic mice

**Fig. 1:**
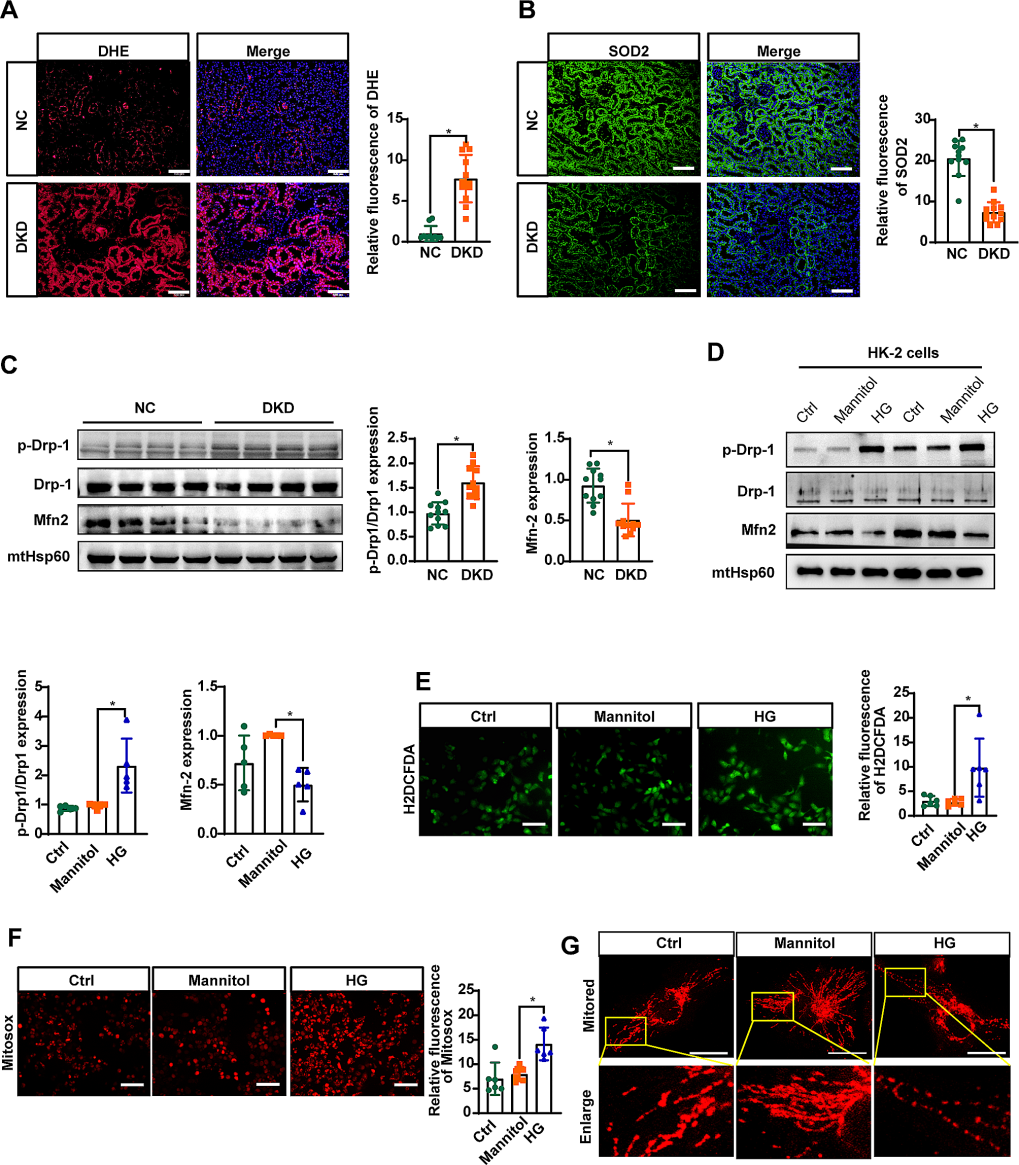
Imbalanced mitochondrial homeostasis is associated with renal proximal tubular injury in DKD. **A** DHE staining was performed to evaluate the production of ROS in the kidneys of the mice. **B** Representative images of SOD2 staining of kidneys from mice. **C** and **D** Representative immunoreactive bands for Drp1, phosphorylated Drp1, Mfn2, and HSP60 in the mitochondrial fractions of the mice (**C**) and HK-2 cells (**D**) as indicated. **E** and **F** H2DCFDA (**E**) and Mitosox (**F**) staining were performed to evaluate the generation of ROS in the cytoplasm and mitochondria of HK-2 cells, respectively. **G** Representative images of MitoTracker Red-stained cells showing mitochondrial morphology in the indicated groups of cells. Scale bar = 100 μm in A and B for fluorescence images, 50 μm for images in E and F, and 20 μm for confocal images in G. For all statistical plots, the data are presented as the means ± SEMs. *P < 0.05

We conducted an immunostaining examination with DHE to investigate ROS production in the kidneys. As illustrated in Fig. 1A, ROS production was significantly greater in the kidneys of diabetic mice than in those of normal control mice. Additionally, the SOD2 immunostaining level in diabetic mice was markedly lower than that in normal control mice, as indicated in Fig. 1B. Previous studies have highlighted the presence of excessive mitochondrial fission and fragmentation during the progression of DKD [24, 25]. Immunoblotting of isolated mitochondria revealed increased levels of phosphorylated Drp1 and decreased levels of Mfn-2 in diabetic mice compared with those in normal controls (Fig. 1D). These protein trends in HK-2 cells cultured with HG align with the findings in diabetic mice. Notably, HG treatment significantly promoted cytosolic and mitochondrial ROS generation in HK-2 cells, as demonstrated by H2DCFDA and Mitosox staining in (Fig. 1E, F). Furthermore, a reduction in mitochondrial length was observed in HK-2 cells exposed to HG conditions for 48 h (Fig. 1G).

### CaMKKβ-mediated protective effect on tubular injury in DKD

**Fig. 2:**
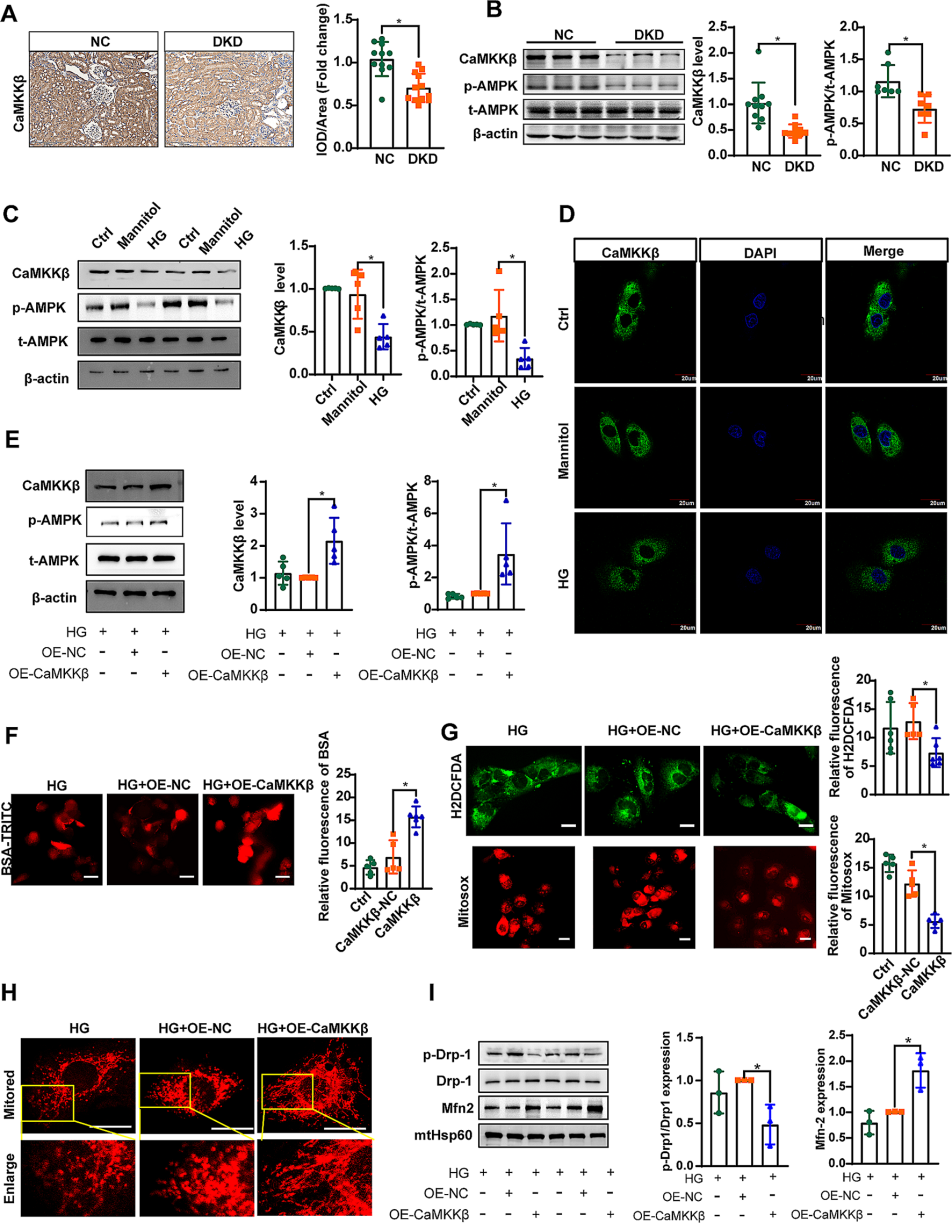
CaMKKβ-mediated protective effect on tubular injury in DKD. **A** IHC staining was performed to evaluate the protein expression of CaMKKβ in kidneys of the mice. Scale bar = 200 μm. **B** and **C** Representative immunoreactive bands for CaMKKβ, AMPK, and phosphorylated AMPK in the groups of mice (**B**) and HK-2 cells (**C**) cultured with HG as indicated. **D** IF staining of CaMKKβ in HK-2 cells cultured with HG for 48 h. Scale bar = 20 μm. **E** Representative immunoreactive bands for CaMKKβ, AMPK, and phosphorylated AMPK in HK-2 cells transfected with CaMKKβ as indicated. **F** IF staining was performed to evaluate the uptake of BSA-TRITC in HK-2 cells. Scale bar = 50 μm. **G** H2DCFDA and Mitosox staining were performed to evaluate the generation of ROS in the cytoplasm and mitochondria of HK-2 cells, respectively. Scale bar = 20 μm. **H** Representative images of MitoTracker Red-stained cells showing mitochondrial morphology in the indicated groups of cells. **I** Representative immunoreactive bands for Drp1, phosphorylated Drp1, Mfn2, and HSP60 in the mitochondrial fraction of HK-2 cells transfected with CaMKKβ as indicated. Scale bar = 20 μm. OE-NC represents the control of CaMKKβ. OE-CaMKKβ represents the overexpression of CaMKKβ. For all the statistical plots, the data are presented as the means ± SEMs. *P < 0.05

The IHC analysis uncovered that CaMKKβ expression was predominantly localized in the renal proximal tubules. Notably, this expression was markedly diminished in the renal tubules of diabetic mice (Fig. 2A). This study also confirmed the decrease in CaMKKβ protein in DKD mice and HG-treated HK-2 cells (Fig. 2B-D). In addition, the expression of p-AMPK/AMPK was significantly decreased under diabetic conditions (Fig. 2B, C). The function of CaMKKβ in HG-induced HK-2 cells was explored in cells transfected with CaMKKβ-overexpressing plasmids, resulting in a notable augmentation of p-AMPK/AMPK expression (Fig. 2E). Additionally, BSA-TRITC uptake in HK-2 cells significantly increased following CaMKKβ overexpression (Fig. 2F). H2DCFDA and Mitosox staining indicated that the overexpression of CaMKKβ suppressed ROS generation (Fig. 2G). Overexpression of CaMKKβ also improved mitochondrial defects by increasing the mitochondrial length and density in HK-2 cells (Fig. 2H). Notably, CaMKKβ overexpression led to a decrease in Drp1 phosphorylation but an increase in Mfn-2 expression (Fig. 2I).

### CaMKKβ and NEDD4L interact directly

**Fig. 3:**
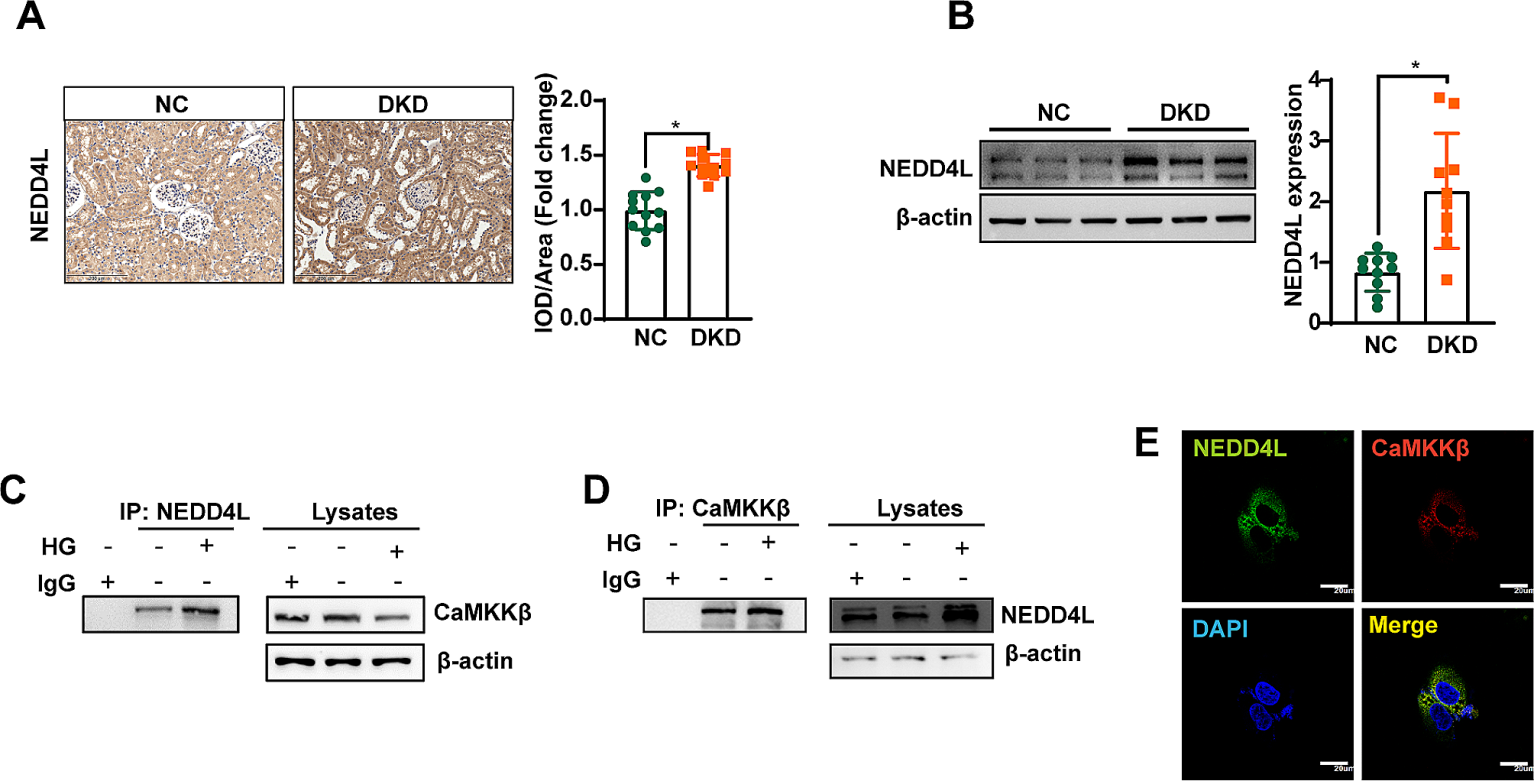
NEDD4L physically interacts with CaMKKβ. **A** IHC staining was performed to evaluate the protein expression of NEDD4L in the kidneys of mice. Scale bar = 200 μm. **B** Representative immunoreactive bands for NEDD4L in groups of mice. For all statistical plots, the data are presented as the means ± SEMs. *P < 0.05. **C** and **D** The interaction between NEDD4L and CaMKKβ was detected in the Co-IP analysis of HK-2 cells. **E** Double staining showed co-localization of NEDD4L with the CaMKKβ in HK-2 cells. Scale bar = 20 μm

Our results and previous research showed that the expression of CaMKKβ is downregulated in DKD [17, 18], however, the specific underlying mechanism remains elusive. The NEDD4 family is characterized by a unique modular domain architecture that contains an amino terminal Ca^2+^ phospholipid binding (C2) domain [26]. NEDD4L is one of the NEDD4 members, and is enriched in the kidney, primarily expressed in renal tubular epithelial cells. NEDD4L has a domain structure with a C2 domain at the N terminus, which can bind calcium [19], and functions as a ligase that facilitates the regulatory processes and binding of diverse membrane proteins for their efficient internalization and turnover. Existing data indicate that NEDD4L is deeply involved in multiple calcium-related pathways [19, 20]. Moreover, depletion of NEDD4L in cells has been associated with heightened mitochondrial membrane potential and the maintenance of mitochondrial fusion status under metabolic stress [21]. In light of these findings, we propose that NEDD4L may modulate mitochondrial homeostasis by influencing CaMKKβ in diabetic renal tubular epithelial cells. To investigate this hypothesis, we initially assessed the expression of NEDD4L in DKD mice through IHC, revealing a prominent localization of NEDD4L in renal proximal tubule cells, with a marked increase in diabetic mice (Fig. 3A), further validated by Western blotting (Fig. 3B). NEDD4L antibodies were used to isolate protein complexes from HK-2 cells, and these complexes were subsequently blotted with CaMKKβ antibodies. As shown in Fig. 3C and D, a physical interaction between NEDD4L and CaMKKβ was detected. In addition, double staining demonstrated a high level of co-localization of NEDD4L with CaMKKβ (Fig. 3E).

### Inhibition of NEDD4L ameliorates protein excretion in diabetic mice

**Fig. 4:**
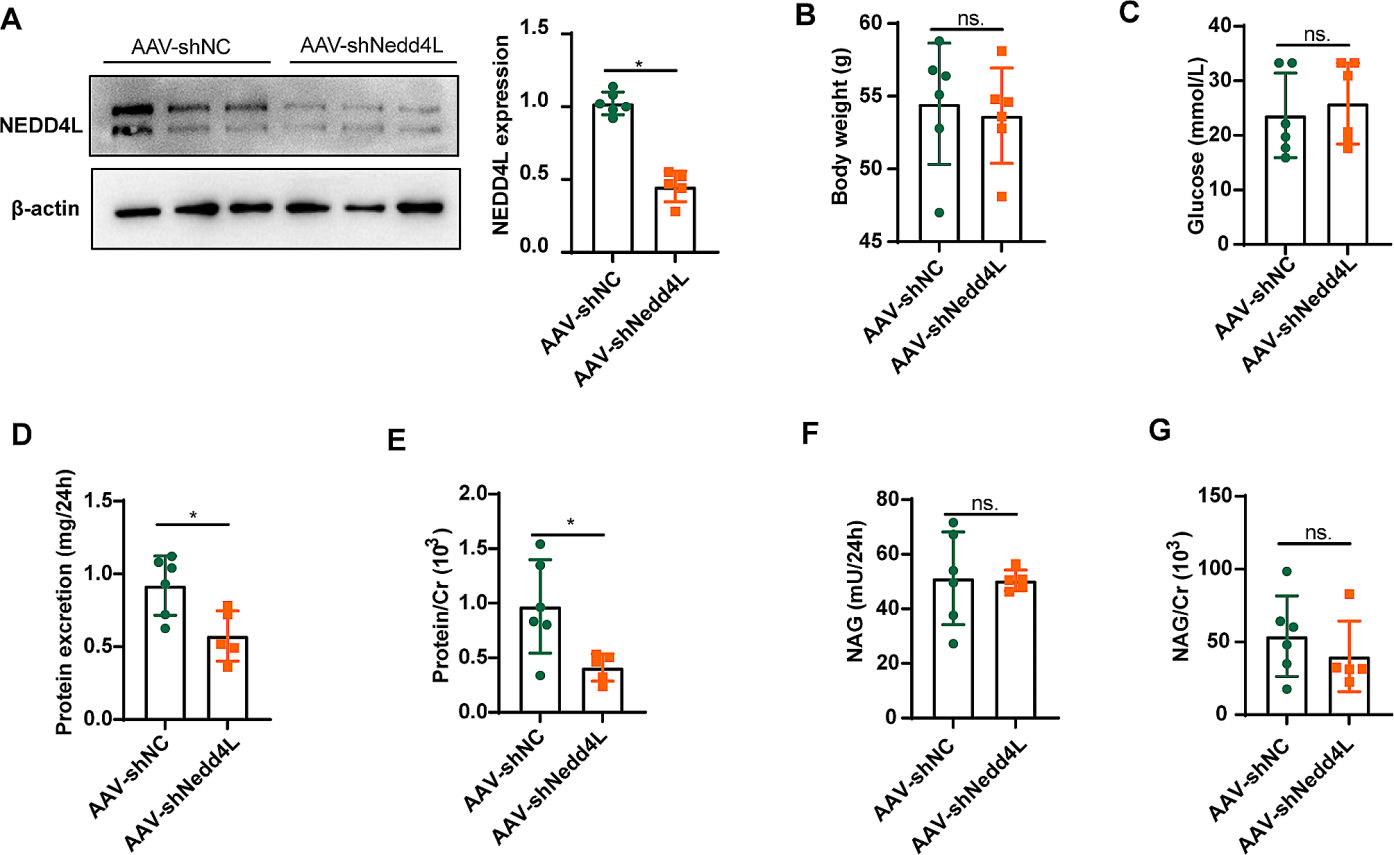
Inhibition of NEDD4L ameliorates protein excretion in diabetic mice. Male db/db mice (8 weeks old) were fed a chow diet for 1 week and then injected with AAV-shNedd4L via the tail vein. **A** NEDD4L knockdown in the kidneys of db/db mice was confirmed by Western blot analysis. **B** and **C** Body weights (**B**) and fasting blood glucose (**C**) were measured in mice 8 weeks after viral infusion. **D** Twenty-four-hour urinary protein excretion was decreased in NEDD4L knockdown mice. **E-G** Protein/Cr (**E**), NAG (**F**), and NAG/Cr (**G**) in 24 h urine samples of the mice. For all the statistical plots, the data are presented as the means ± SEMs. ns. indicates no significance; *P < 0.05

db/db mice injected with AAV were used to investigate the involvement of NEDD4L in DKD development. Following injection of AAV-shNedd4L, the expression of NEDD4L in the renal cortex of db/db mice significantly decreased (Fig. 4A). Body weight and blood glucose levels did not significantly change across all groups (Fig. 4B, C). Protein excretion was significantly decreased in db/db mice injected with AAV-shNedd4L (Fig. 4D, E). Additionally, NAG and NAG/Cr also showed a decreasing trend, but the difference was not statistically significant (Fig. 4F, G). Previous studies have indicated that NEDD4L may impact the balance of potassium and sodium in the kidney [27, 28]. Furthermore, an analysis of potassium, sodium, and other ions in both blood and urine samples revealed no significant variations among the groups, as presented in Supplementary Fig. 1.

### Inhibition of NEDD4L improves mitochondrial homeostasis in diabetic mice

**Fig. 5:**
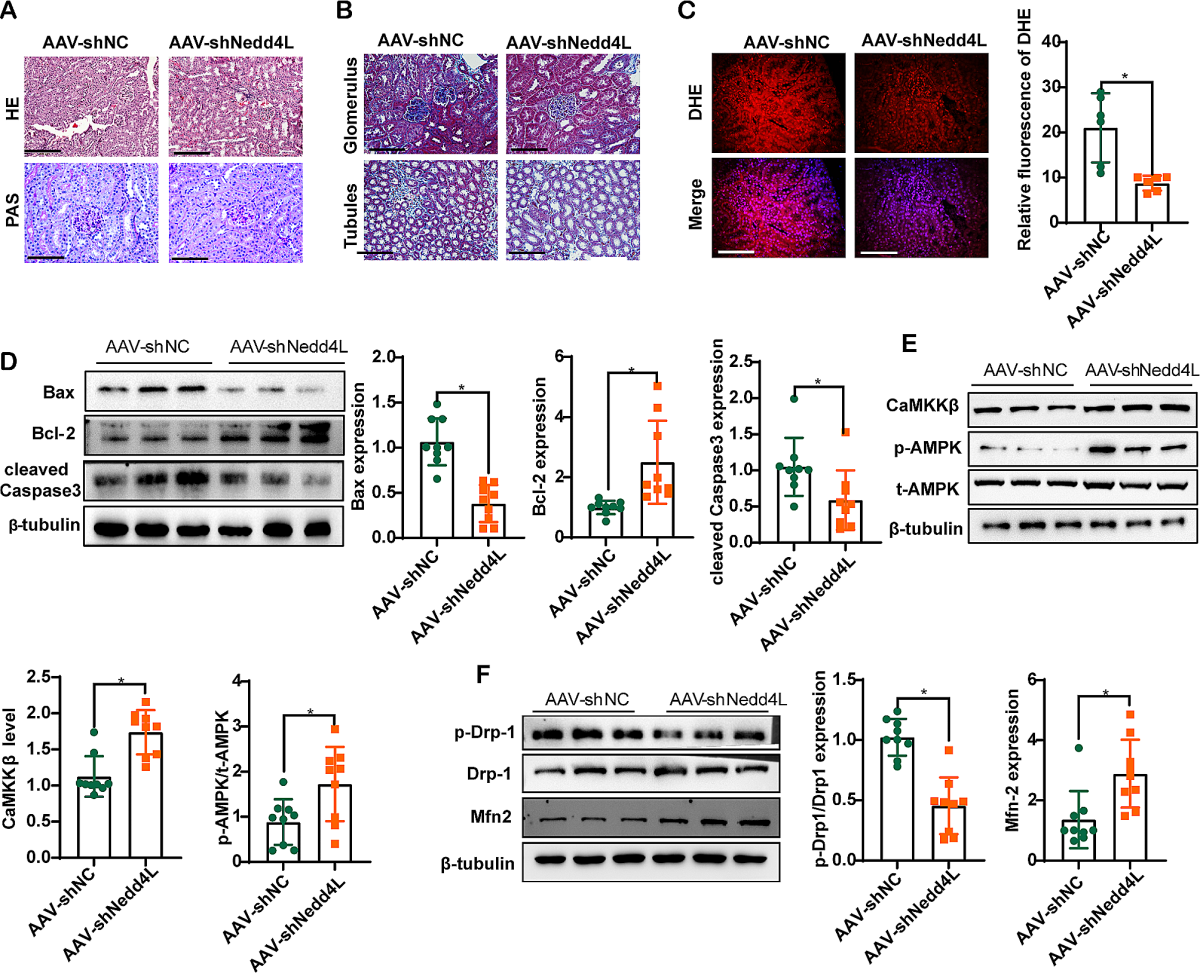
Inhibition of NEDD4L ameliorates oxidative stress and mitochondrial dysfunction in diabetic mice. **A** Representative images of HE and PAS staining of kidney sections. **B** Representative images of Masson staining of kidney sections. **C** Representative images of DHE-stained kidney sections showing oxidative stress in the mice. A-C: Scale bar = 200 μm. **D** Representative immunoreactive bands for Bax, Bcl-2, and cleaved Caspase-3 in the groups of mice. **E** Representative immunoreactive bands for CaMKKβ, phosphorylated AMPK, and total AMPK in the groups of mice. **F** Representative immunoreactive bands for Drp1, phosphorylated Drp1, Mfn2, and HSP60 in different groups of mice, as indicated. For all the statistical plots, the data are presented as the means ± SEMs. *P < 0.05

The db/db mice injected with the negative control exhibited significant glomerular enlargement, mesangial matrix expansion, tubular hypertrophy, dilation, and disruption of the tubular basement membrane. Masson staining further revealed a notable increase in tubulointerstitial fibrosis in diabetic mice (Fig. 5A, B). However, diabetic mice injected with AAV-shNedd4L showed significant improvements compared to those in the negative control group (Fig. 5A, B). DHE staining demonstrated that AAV-shNedd4L alleviated oxidative stress in diabetic mice (Fig. 5C). Compared to the negative control diabetic mice, shNedd4L-treated diabetic mice exhibited reduced renal apoptosis, as indicated by decreased expression of Bax/Bcl2 and cleaved Caspase3 (Fig. 5D). Additionally, CaMKKβ/AMPK signaling was upregulated following shNedd4L treatment (Fig. 5E). Immunoblotting of isolated mitochondria revealed decreased phosphorylation levels of Drp1 and increased levels of Mfn-2 in the AAV-shNedd4L db/db mice as compared with the negative controls (Fig. 5F).

### Silence of NEDD4L prevents HG-induced mitochondrial dysfunction in HK-2 cells

**Fig. 6:**
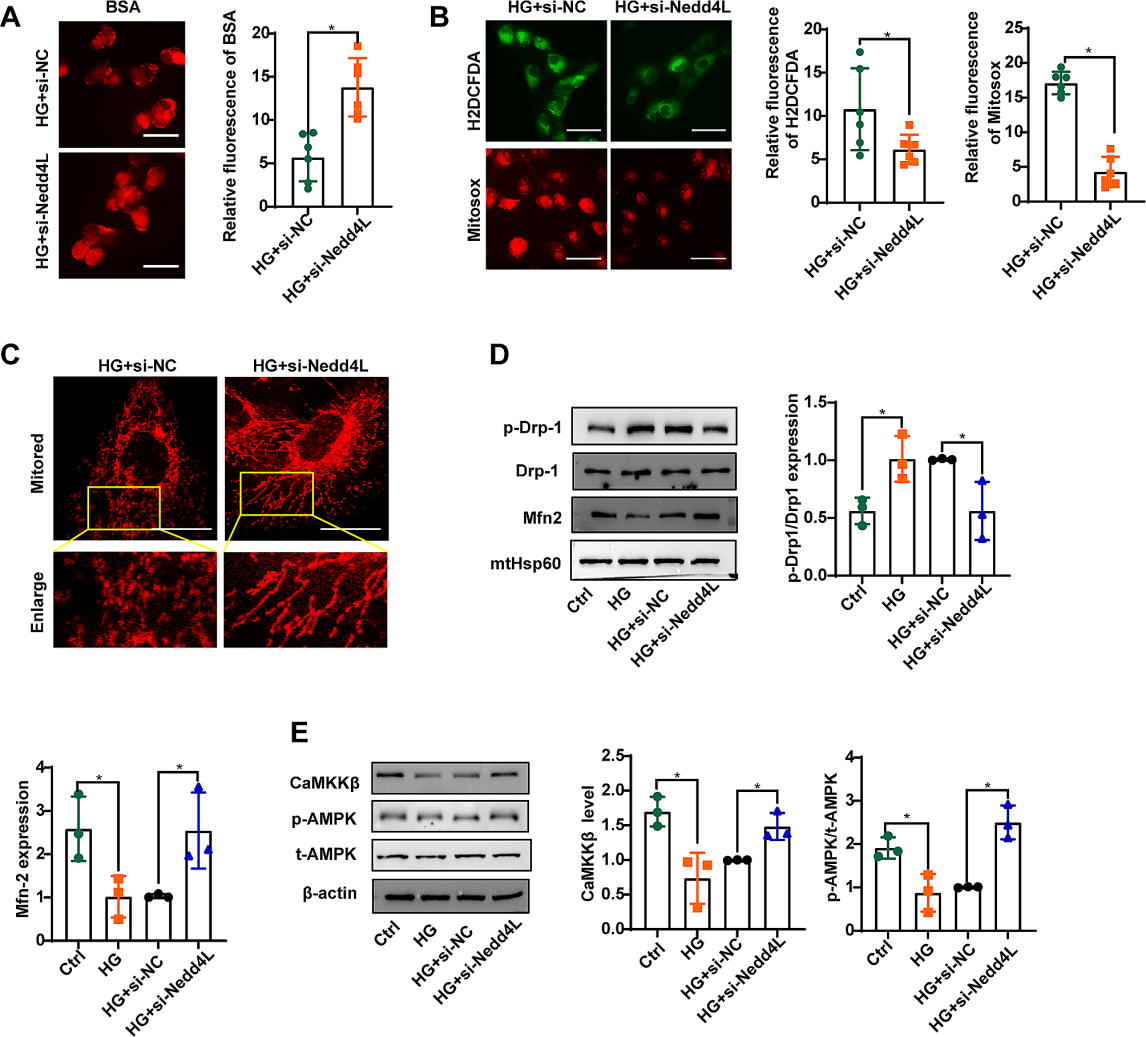
Silencing NEDD4L prevents HG-induced mitochondrial dysfunction in HK-2 cells. **A** IF staining was performed to evaluate the uptake of BSA-TRITC in the experiments. **B** H2DCFDA and Mitosox staining were performed to evaluate the generation of ROS in the cytoplasm and mitochondria of HK-2 cells, respectively. Scale bar = 50 μm. **C** Representative images of MitoTracker Red-stained cells showing mitochondrial morphology in the indicated groups of cells. Scale bar = 20 μm. **D** Representative immunoreactive bands for Drp1, phosphorylated Drp1, Mfn2, and HSP60 in the mitochondrial fraction of HK-2 cells transfected with Nedd4L-siRNA as indicated. **E** Representative immunoreactive bands for CaMKKβ, AMPK, and phosphorylated AMPK in HK-2 cells transfected with Nedd4L-siRNA as indicated. For all the statistical plots, the data are presented as the means ± SEMs. *P < 0.05

The uptake of BSA-TRITC in HG-treated HK-2 cells was significantly enhanced following the silencing of NEDD4L (Fig. 6A). Staining with H2DCFDA and Mitosox revealed a marked suppression of ROS generation by si-Nedd4L (Fig. 6B). In vitro, si-Nedd4L improved mitochondrial defects, leading to increased mitochondrial length and density in HK-2 cells (Fig. 6C). Notably, si-Nedd4L significantly reduced the phosphorylation of Drp1 but increased Mfn-2 expression (Fig. 6D). Furthermore, transfection with si-Nedd4L rescued CaMKKβ expression in HK-2 cells, as confirmed by Western blot analysis (Fig. 6E). The ratio of phosphorylated AMPK to total AMPK (p-AMPK/AMPK) was also notably elevated post NEDD4L knockdown (Fig. 6E).

### NEDD4L leads to impaired mitochondrial homeostasis via CaMKKβ expression inhibition

**Fig. 7:**
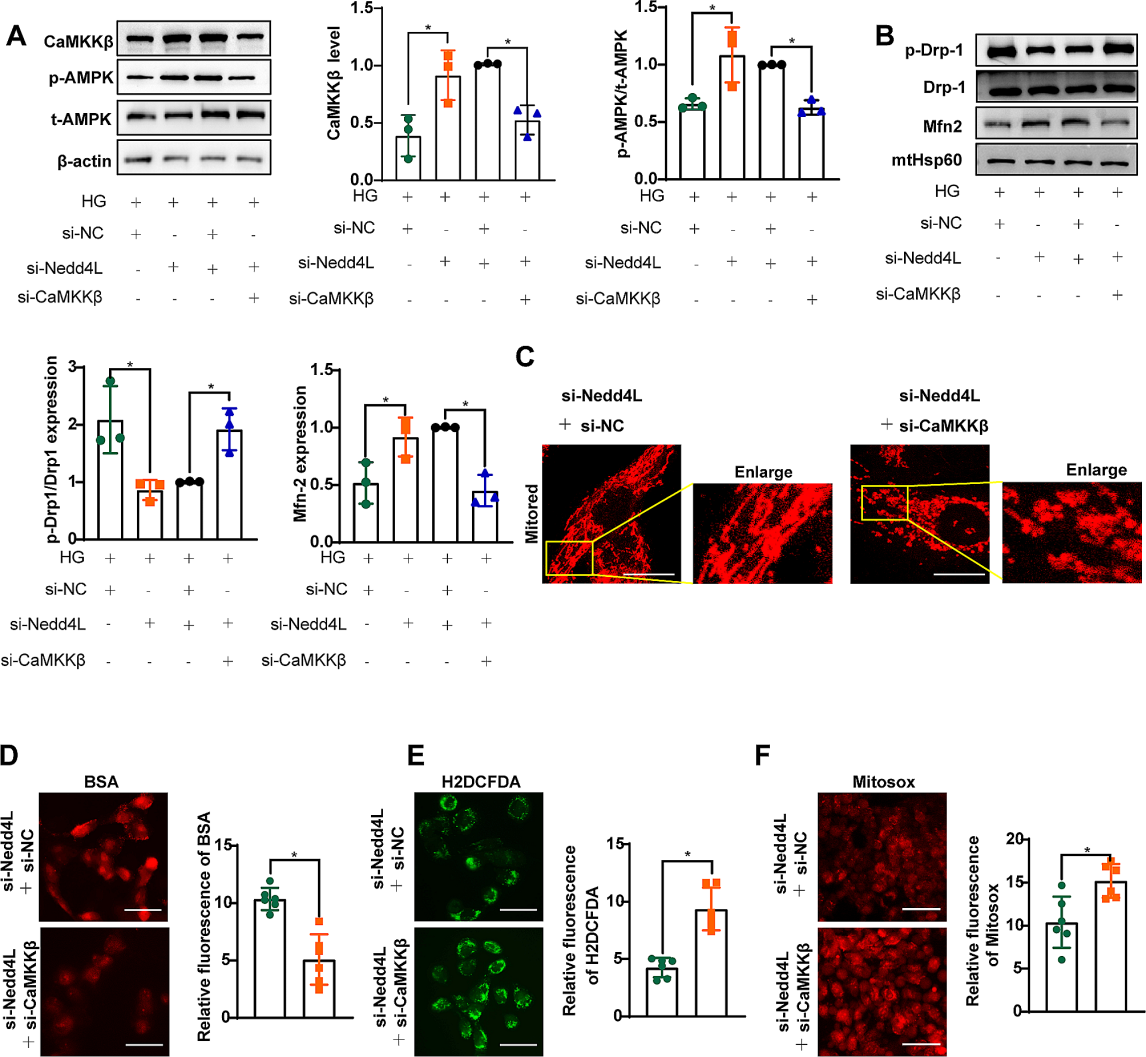
Knockdown of NEDD4L contributes to HK-2 cell function via CaMKKβ/AMPK pathway. **A** Representative immunoreactive bands for CaMKKβ, AMPK, and phosphorylated AMPK in HK-2 cells cotransfected with Nedd4L-siRNA and CaMKKβ-siRNA as indicated. **B** Representative immunoreactive bands for Drp1, phosphorylated Drp1, Mfn2, and HSP60 in the mitochondrial fraction of HK-2 cells, as indicated. **C** MitoTracker Red staining showing mitochondrial morphology in the indicated groups of cells. Scale bar = 20 μm. **D** IF staining was performed to evaluate the uptake of BSA-TRITC in the indicated groups of cells. Scale bar = 50 μm. **E** and **F** H2DCFDA (E) and Mitosox (F) staining were performed to evaluate the generation of ROS in the cytoplasm and mitochondria of HK-2 cells, respectively. Scale bar = 50 μm. For all the statistical plots, the data are presented as the means ± SEMs. *P < 0.05

In HK-2 cells treated with HG, the expression of CaMKKβ/AMPK pathway was upregulated by NEDD4L-siRNA, however, this upregulation was reversed by CaMKKβ knockdown (Fig. 7A). Moreover, mitochondrial fission induced by HG was alleviated after NEDD4L knockdown, but this improvement was also abolished by CaMKKβ knockdown (Fig. 7B, C). Additionally, CaMKKβ-siRNA reversed the improvements in BSA uptake and oxidative stress observed following treatment with NEDD4L-siRNA (Fig. 7D-F). These results collectively suggest that NEDD4L may impact mitochondrial function through its regulation of the CaMKKβ pathway.

### NEDD4L negatively regulates the stability of CaMKKβ

**Fig. 8:**
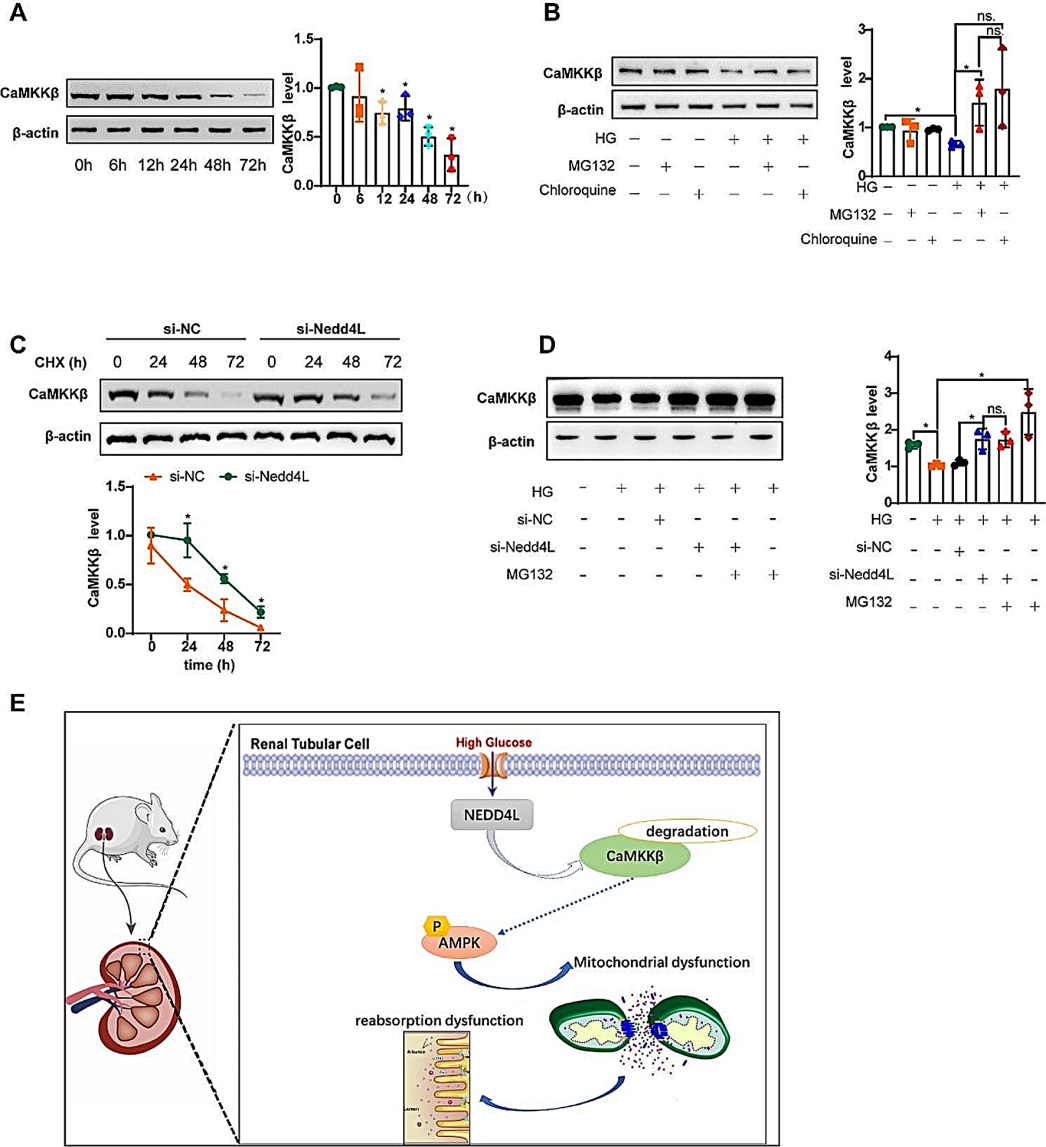
NEDD4L promotes protein degradation of CaMKKβ. **A** Representative immunoreactive bands for CaMKKβ in HK-2 cells exposed to HG as indicated. **B** Representative immunoreactive bands for CaMKKβ in HK-2 cells treated with 10 µM MG132 or 20 µM chloroquine. **C** HK-2 cells cultured with 25.5mM glucose were transfected with si-NC or si-Nedd4L for 24 h. After coculture with 50 mM cycloheximide (CHX) for 0, 24, 48, or 72 h, western blot analysis was performed, and the relative CaMKKβ expression was calculated. **D** HK-2 cells were transfected with si-NC or si-Nedd4L for 24 h and then treated with or without MG132 for another 48 h. For all the statistical plots, the data are presented as the means ± SEMs. *P < 0.05; ns. indicates no significance. **E** The expression of NEDD4L was found to be elevated in DKD, leading to the facilitation of CaMKKβ degradation, thereby disrupting mitochondrial homeostasis

The expression of endogenous CaMKKβ was significantly decreased under diabetic conditions (Fig. 2A-C). Furthermore, treatment with HG led to a time-dependent inhibition of CaMKKβ expression (Fig. 8A). To further clarify whether HG affects CaMKKβ stability through the proteasome or lysosome pathway, we treated cells with the proteasome inhibitor MG132 or the lysosome inhibitor chloroquine. The decrease in CaMKKβ levels caused by HG could be effectively reversed by MG132 but not by chloroquine, suggesting that proteasome-mediated degradation is the primary cause of the HG-induced decrease in CaMKKβ levels (Fig. 8B). Furthermore, the deletion of NEDD4L prevented the rapid decrease in CaMKKβ protein levels after exposure to HG (Fig. 8C). Deletion of NEDD4L also reversed the decrease in total CaMKKβ protein in HK-2 cells exposed to HG, which was not different from the effect of MG132 (Fig. 8D), indicating that Nedd4L-siRNA and MG132 inhibit different steps of the same CaMKKβ degradation pathway under HG conditions. In summary, the presented findings provide substantial evidence that dysregulated NEDD4L disrupts mitochondrial homeostasis by adversely modulating CaMKKβ in DKD (Fig. 8E).

## Discussion

In this study, we found that increased NEDD4L expression in renal proximal tubules resulted in reduced BSA uptake, disrupted mitochondrial homeostasis, and elevated oxidative stress. Conversely, inhibition of NEDD4L effectively alleviated DKD. Our findings also revealed a significant association between the CaMKKβ/AMPK pathway and mitochondrial function as well as apoptosis. Specifically, we observed a downregulation of the CaMKKβ/AMPK pathway under diabetic conditions and demonstrated the protective role of CaMKKβ overexpression in HK-2 cells. Additionally, our study revealed that NEDD4L disrupts mitochondrial homeostasis by promoting the degradation of CaMKKβ in DKD. These results suggest a potential mechanism by which NEDD4L contributes to renal proximal tubule dysfunction in DKD patients and highlight the importance of the CaMKKβ/AMPK pathway in mitigating the effects of NEDD4L. Further exploration of these pathways may offer insights into novel therapeutic targets for DKD.

Ca^2+^ is an important and pervasive second messenger that also maintains the function of mitochondria [29]. CaMKKβ/AMPK signaling plays an important role in regulating mitochondrial homeostasis. Prior research has demonstrated that Aβ42 oligomers trigger mitochondrial fragmentation and synaptotoxicity through the overactivation of CaMKKβ-AMPK [29]. CaMKKβ/AMPK-mediated autophagy participates in podocyte injury under diabetic conditions [17, 18]. However, the role of the CaMKKβ/AMPK pathway in regulating diabetic tubular epithelial cells remains unclear. Our data showed that the CaMKKβ/AMPK pathway was suppressed in DKD, resulted in a reduction of mitochondrial fragmentation and oxidative stress, while enhancing the uptake of BSA in HK-2 cells induced by HG. The above results demonstrated the protective role of CaMKKβ/AMPK signaling in HK-2 cell function.

We sought to investigate the downregulation of CaMKKβ under diabetic conditions and conducted MG-132 and chloroquine assays. Our findings indicate that proteasome-mediated degradation is the primary cause of HG-induced decreases in CaMKKβ levels. The NEDD4 family members are characterized by a unique modular domain architecture containing an N-terminal C2 domain [26]. NEDD4L, a member of this family closely associated with Ca^2+^ signaling [30, 31], is involved in the regulation of protein stability [21, 32]. Our findings revealed a negative relationship between NEDD4L and CaMKKβ levels. Deletion of NEDD4L prevented the rapid degradation of the CaMKKβ protein in response to HG exposure. Furthermore, in vitro studies indicated that abnormal expression of NEDD4L negatively regulates the protein stability of CaMKKβ.

Our study revealed a significant elevation in the expression of NEDD4L specifically within the kidney, with a predominant localization in the renal proximal tubules. Utilizing AAV-shNedd4L for NEDD4L knockdown demonstrated a protective effect against DKD. A previous study showed that mice deficient in NEDD4L, specifically in the renal tubules (Nedd4L Ksp1.3), developed mild kidney disease due to the upregulation of ENaC [33]. Additionally, NEDD4L likely plays a role in maintaining the balance of potassium and sodium in the kidney [27, 28]. The aforementioned studies strengthened the understanding of the impact of NEDD4L on the kidney, primarily through its regulation of Na^+^ and K^+^ absorption. However, our current research demonstrated that knockdown of NEDD4L in the kidneys of db/db mice using AAV did not disrupt the balance of Na^+^ and K^+^, potentially accounting for the discrepancies with previous findings. Nevertheless, further investigations are warranted to comprehensively elucidate the role of NEDD4L.

The role of mitochondrial integrity in kidney tubular cells is crucial for the efficient production of ATP and oxidative phosphorylation. It has been suggested that enhancing the balance of mitochondria in renal proximal tubular cells could be a viable strategy for treating and preventing DKD. In our research, we investigated the influence of NEDD4L on mitochondrial dynamics and found that NEDD4L may contribute to mitochondrial fission, a finding that aligns with that of a previous study [21]. The aforementioned study demonstrated that cells with reduced NEDD4L exhibited elongated mitochondria in a more fused state than did control cells [21]. In our research, we observed that silencing NEDD4L attenuated mitochondrial fission in HG-induced HK-2 cells. However, the effect of si-NEDD4L was nullified upon knockdown of CaMKKβ. These findings suggest that NEDD4L potentially influences DKD by modulating CaMKKβ and its downstream signaling pathways.

In conclusion, these findings highlight the significance of aberrant NEDD4L expression in disrupting mitochondrial homeostasis by downregulating CaMKKβ in diabetic kidney disease. This insight offers a potential therapeutic strategy targeting NEDD4L and CaMKKβ to preserve renal tubular function in the context of diabetes. The potential for therapeutic intervention in this pathway could hold promise for the treatment of DKD.

### Electronic supplementary material

Below is the link to the electronic supplementary material.

Supplementary Material 1

## Data Availability

The datasets used in the present study are available from the first author upon reasonable request.

## Abbreviations

AAV: adeno-associated virus
AMPK: 5’ AMP-activated kinase
BSA: bovine serum albumin
Ca^2+^: calcium ion
CaMKKβ: Calmodulin-dependent protein kinaseβ
DHE: Dihydroethidium
DKD: Diabetic Kidney Disease
HE: Hematoxylin and Eosin
HG: high glucose
IHC: immunohistochemical
IP: immunoprecipitation
mito-ROS: mitochondrial ROS
PAS: Periodic acid-Schiff
