# Supplementary material for: Aberrant expression of NEDD4L disrupts mitochondrial homeostasis by downregulating CaMKKβ in diabetic kidney disease
Source: J Transl Med. 2024 May 16;22:465. doi: 10.1186/s12967-024-05207-6 (PMC11100153; doi:10.1186/s12967-024-05207-6)
Supplement: Supplementary file 1 — Supplementary Material 1 [file 12967_2024_5207_MOESM1_ESM.docx]

Supplementary Fig1


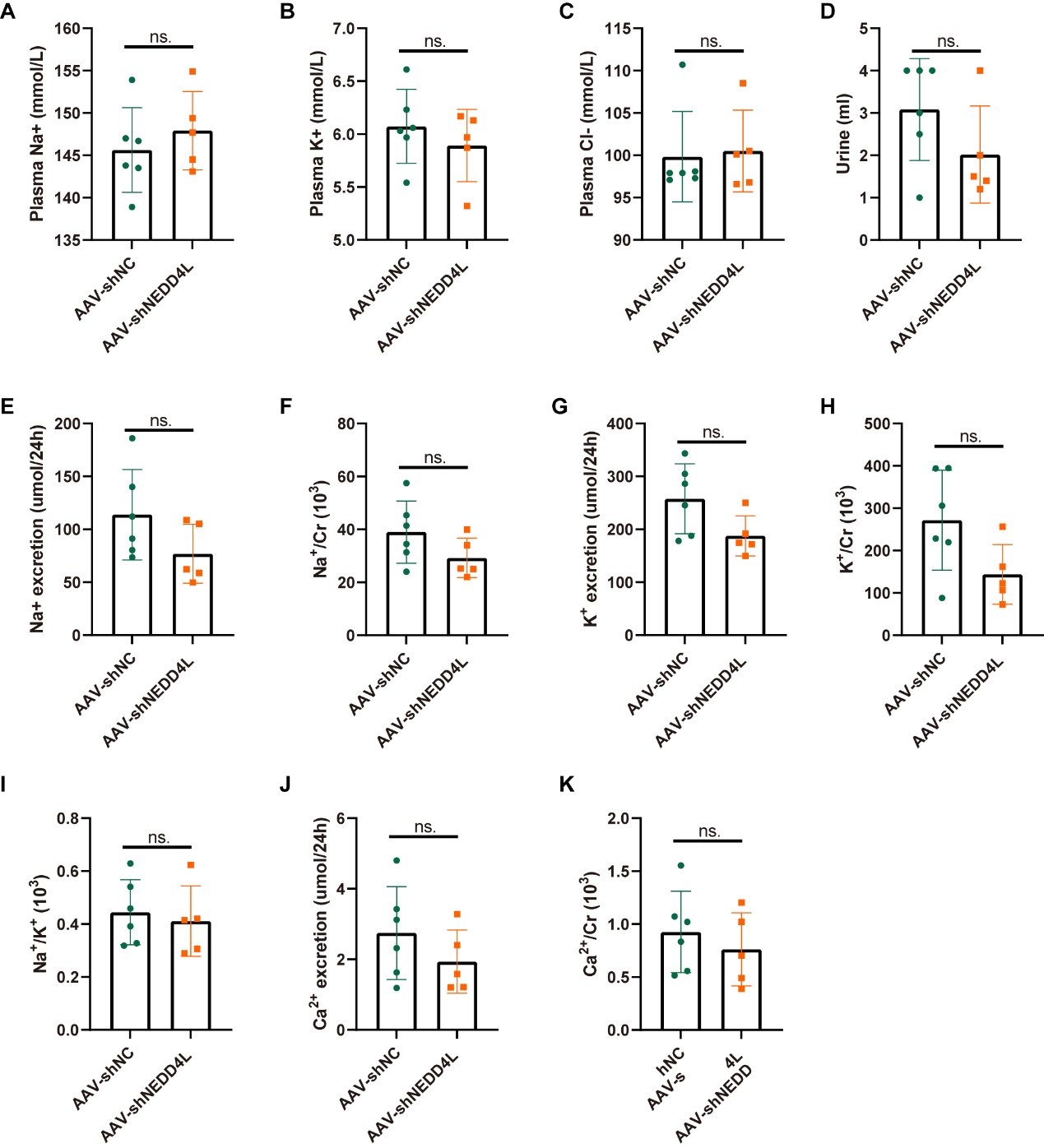


sFig1 Sh-NEDD4L had no effect on the iron levels in blood or urine. **A-C** Plasma Na^+^ (A), K^+^ (B), and Cl^-^ (C) levels were tested in the mouse. **D** The 24-hour urine volume of the mouse. **E-K** The levels of Na^+^ excretion (E), Na^+^/ Cr (F), K^+^ excretion (G), K^+^/ Cr (H), Na^+^/ K^+^ (I), Ca^2+^ excretion (J), and Ca^2+^ / Cr (K) in 24-hour urine were assessed. ns, no significant
